# Both Gender and Agonistic Experience Affect Perceived Pain during the Cold Pressor Test

**DOI:** 10.3390/ijerph19042336

**Published:** 2022-02-18

**Authors:** Pierluigi Diotaiuti, Stefano Corrado, Stefania Mancone, Marco Palombo, Angelo Rodio, Lavinia Falese, Elisa Langiano, Thaìs Cristina Siqueira, Alexandro Andrade

**Affiliations:** 1 Department of Human Sciences, Society and Health, University of Cassino and Southern Lazio, 03043 Cassino, Italy; stefano.corrado@unicas.it (S.C.); s.mancone@unicas.it (S.M.); marco.palombo@unicas.it (M.P.); a.rodio@unicas.it (A.R.); l.falese@unicas.it (L.F.); langiano@unicas.it (E.L.); 2 Health and Sports Science Center, Department of Physical Education, CEFID, Santa Catarina State University, Florianópolis 88035-901, Brazil; thais.siqueira@udesc.br (T.C.S.); alexandro.andrade@udesc.br (A.A.)

**Keywords:** athletes, gender, agonistic experience, pain tolerance, cold pressor test, temporal summation of pain

## Abstract

Background. Differences in pain perception in athletes have recently been highlighted in the literature. Objectives. To compare gender ratings of perceived pain in athletes with low and high agonistic experiences (N = 200) using the Cold Pressor Test (CPT). Methods. A three-way repeated measures ANOVA to assess both the effects of the athletes’ gender and lower vs. higher agonistic experiences in the intensity of perceived pain at the beginning of the cold box hand immersion (L_0_) and after a 90 s interval (L_1_). Results. There was a statistically significant interaction effect between the level of the agonistic experience and gender in the two moments: *p* < 0.001; η_p_^2^ = 0.266; F(1,49) = 9.771. Simple main effects analysis showed a significative difference for females at L_0_: F(1,99) = 93.567, *p* < 0.025, partial η^2^ = 0.302) and for males at L_1_: F(1,99) = 173.420, *p* < 0.025, partial η^2^ = 0.666. At the initial moment of CPT, the female athletes showed significantly higher perceived intensity than males, regardless of their experience level. After a 90 s interval, a significantly lower pain perception effect associated with the increased competitive experience of male athletes was observed. Female athletes did not appear to benefit from the experience effect on their pain tolerance. Conclusions. The study confirmed a significant difference in pain perception associated with the athletes’ gender and agonistic experience. Separate explanations related to the pattern of pain inhibition and the acquired reduction in pain sensitivity are reported.

## 1. Introduction

Several studies have shown that athletes generally perceive less pain than non-athletes [1,2], and current contributions provide evidence that antinociceptive effects are dependent on the intensity of exercise [3]. Research has also shown gender differences in pain perception [4,5]. Emerging evidence suggests that genotype and endogenous opioid functioning play a causal role in these disparities, and considerable literature implicates sex hormones as factors influencing pain sensitivity [6,7]. Several studies have investigated the relationship between gender and pressure pain sensitivity, and most results found that females have significantly lower pain thresholds than males [8,9,10]. One possible mechanism contributing to gender differences in pain sensitivity might be differences in the activation of conditioned pain modulation (CPM), namely, the decreased sensitivity response to a conditioning pain stimulus (e.g., pressure, cold water or ischemia) [11,12].

Gender differences could also be explained with the help of a multifactorial model of pain, according to which biological, psychological and social factors interact in the perception of pain [13,14]. It appears that gender-role expectations of pain do play a part in determining an individual’s pain report and may be contributing to the gender differences in the laboratory setting [15].

Although sport-related research on pain is scarce, within the context of athletic performance a different set of social learning factors may be operating [16,17,18]. A recent investigation using the cold-water pressor test examined differences in pain perception between university athletes and sedentary controls, and it has been found that the athletes reported less pain than the sedentary individuals, with men reporting less pain than women [19]. Regression analyses revealed that catastrophizing accounted for differences between men and women as to pain perception. While for men it appears that anxiety influences the perception of pain, women tend more towards a catastrophic approach to pain, which includes rumination, which prevents them from suppressing or diverting attention from pain-related thoughts to focus on other things, a tendency to exaggerate the unpleasantness of pain and the expectation of negative outcomes and a sense of helplessness, which reflects the inability to do anything effective to manage the moment. The incidence of catastrophic disposition and self-efficacy evaluation on pain perception has also been recently corroborated in further studies [20,21].

Further research has shown that for men, the stimulus of competition appears to be decisive in mobilising the body’s pain-relieving resources, whereas women could achieve a comparable pain-relieving effect through exercise alone [22].

In any case, as athletes progress in training and experience, they seem to be able to tolerate more pain than their non-trained counterparts [23]. The contribution of competitive experience in pain tolerance can be traced back to three relevant aspects: increased athletic work, the strengthening of coping strategies and greater social reinforcement. With regard to the first element, it is plausible that athletes with greater experience (also understood in terms of the competitive level achieved: regional, national or international) accumulate greater volumes of training over time. These evolve in terms of frequency, articulation and intensity, the greater number of competitions faced and the greater familiarity with painful experiences such as injuries. All this can significantly influence the modulatory process of perceived pain at a neurophysiological level [24,25,26].

The second element considered is the progressive strengthening of coping strategies. Some athletes have natural predispositions to such higher psychological resistance to pain, but most sportspeople need to develop it, identifying which coping strategies they could use to maintain their physical involvement despite being in pain. Such strategies include distraction from pain, praying, reinterpreting pain sensations, ignoring pain and pain catastrophizing [27]. Recent studies have attributed this greater adaptation to pain tolerance to the progressive experience of control of the situation [28,29,30] and to the development of a strong awareness that allows perceived painful sensations to be ignored [31,32,33,34,35].

With regard to the third element, a greater competitive experience provides the athlete with progressive social reinforcement (from the context of reference) and awareness of competence. Athletes who have publicly demonstrated that they have overcome difficult, burdensome and painful challenges increases their level of self-esteem and personal self-efficacy, feeling increasingly confident in the possibility of successfully dealing with further painful experiences [36,37]. The influence of the expectations of the sporting environment must also be pointed out: being immersed in the elite sport culture encourages the normalisation of pain and injury, and it is widely accepted that athletes, regardless of gender, internalise pain and injury as ‘normal’ in sport [38,39].

By adopting a multidimensional model of pain as a theoretical framework, the component factors of the model (sensory-discriminative, affective-motivational, cognitive-evaluative) are not considered as independent, but rather, they interact with one another [13,14]. The subsequent explanatory hypotheses of subjective pain sensitivity in athletes should also consistently focus on the interaction of the component variables, rather than on the identification of a dominant factor (be it physiological, psychological or social). For these reasons, our work is aimed at testing the interaction between gender and the components of the athlete’s experience as explanatory of variation in perceived pain.

A relevant aspect of the pain experience is the possible perception of a progressive increase in the intensity of the stimulus over time. This evaluation is usually conducted through the paradigm of the Temporal Summation of Pain (TSP). The TSP paradigm is the experimental model that is most frequently used to study endogenous excitatory pain mechanisms (e.g., central sensitization) [40,41]. Although the method has been used extensively in clinical settings with both patients and healthy subjects [42,43,44,45,46], a few studies have employed repetitive (thermal and mechanical) noxious stimuli with athletes to more specifically assess individual and group differences in their tolerance of chronic pain over time [47,48,49]. To the best of our knowledge, there are no previous studies that have evaluated the interaction between gender and competitive experience on the athlete’s perception of pain using CPT.

## 2. Materials and Methods

### 2.1. Study Design

This study featured a comparative balanced cross-sectional approach.

### 2.2. Participants

The population was characterized by university student athletes who attend the University of Cassino and Southern Lazio. The sample was recruited in a non-probabilistic way through an open invitation to the entire study population disseminated through student social channels and with the support of the University Sports Centre (CUS). The students were informed that they would participate in a laboratory study to measure subjective sensitivity to cold. Inclusion criteria were: (1) age range of 18–28 years, (2) competitive athletes of individual or team disciplines at the regional or national level. Exclusion criteria were: (1) inability to understand and follow instructions in verbal and written Italian; (2) any health conditions potentially causing sensory deficits, such as diabetes mellitus or neurological disorders; (3) any history of chemotherapy; (4) the current assumption of medication that can affect sensation; and (5) a current pregnancy. Students interested in participating were invited to book using a special link in the announcement, which, once opened, allowed them to enter their contact details, also indicating their gender, age, competition level (national/regional), number of years practising the sport, number of competitions in the last three years, number of training sessions per week and average duration of training. The researchers then organised and informed the students of the specific date and time at which they would be summoned to the laboratory. In relation to the study design, as the aim was to compare perceived pain on the CPT test in a higher agonistic experience and a lower agonistic experience group of athletes while also assessing the gender difference, the list and the information provided by those booked into the study was subjected to a preliminary analysis in order to apply useful criteria to divide the participants into a high experience group and a lower experience group. Using a non-hierarchical cluster analysis, the classification variables considered between the two groups were: their competition level (national/regional), the number of years of sport experience, the number of competitions in the last three years, the number of weekly training sessions and the average duration of training. Considering the decreasing measure of the distance of the case from the centroid, 50 males and 50 females were selected from the list in the 2 groups, for a total of 200 participants (100 higher experience, 100 lower experience). Selected participants were asked to limit intake of caffeine, alcohol and any medication causing sleepiness or analgesia for 24-h prior to the testing session. The procedure was explained, and written informed consent was obtained before data collection commenced.

### 2.3. Procedures

The study was approved by the Institutional Review Board of the University of Cassino and Southern Lazio (IRB_SUSS_07:24/03/19). The participants (all volunteers) were each summoned to the laboratory in morning sessions (in the 9 a.m.–12 a.m. time slot). They (a) received information on the study from the investigators; (b) provided informed consent to participate and were informed about the safety on the scientific and aggregate use of the data provided, in accordance with the Declaration of Helsinki; (c) completed a preliminary questionnaire for the collection of demographic data; and (d) carried out the session of the cold pressor test (CPT). The CPT was adopted as a method to induce and measure variations in pain perception. Participants were given the option to discontinue the test at any time in which the pain sensation was deemed intolerable. Before the execution of the CPT, the protocol included an acclimatization phase in which the participants were asked to immerse their non-dominant hand inside a basin containing three litres of water at room temperature for two minutes, both in order to accustom the hand to low temperatures and to make the basal temperatures uniform for the participants before the test.

The participant was then asked to place his/her non-dominant hand and wrist in 8 °C water in a 13-L plexiglass container connected with a circulating water bath (Julabo PF40-HE) and maintain it there as long as he/she could or until the maximum time limit of 90 s was reached. For safety reasons, the test was terminated after 1.5 min (90 s) if the participant had not already removed his/her hand. Although the maximum immersion limit of 3–5 min is normally applied to healthy adults [50,51,52,53], the test limit of 1.5 min was chosen to limit the risk of tissue injury.

The perception of the pain component was evaluated by scoring on a 10-interval numerical graduation scale (0 = no perceived pain, 10 = maximum perceived pain). In our case, as pain stimulus, the subject was required to immerse their non-dominant hand and indicate with their index finger of the other hand the progression of painful perception from the initial to the final moment of the test (90 s). However, all participants were given the option to withdraw their hand and interrupt the test when the discomfort was considered unbearable. The experimenter recorded the starting and ending time with a stopwatch, as well as the time of any voluntary withdrawal of the subject from the test. In case of withdrawal, the participant was given the highest score on the final pain perception record. A portable camera placed on a support behind the subject allowed the recording of the values of the progression of perception indicated on paper on a scale from 1 to 10. The stability of the water temperature in the tank was also controlled with the help of an internal thermometer and using a KK Moon IR external infrared thermometer device. A maximum temperature fluctuation of 3 °C was allowed in the tank.

### 2.4. Instruments

The CPT was performed using a Julabo Termocriostat (CF40-HE, Julabo GmbH, Seelbach, Germany), an external infrared thermometer (IR, KKmoon, Shenzhen, China) and an LCD thermometer with suction cup (Ueetek, Shenzhen, China). The experiment was also recorded using a video camera (Camcorder FHD 1080P, Panasonic, Kadoma, Japan).

### 2.5. Statistical Analysis

Statistical analyses were performed using the package SPSS (IBM) version 26. A non-hierarchical cluster analysis was used to divide participants into two groups of different experience considering established classification variables such as number of weekly training sessions, average duration of training sessions, number of competitions in the last three years, competitive level and total years of practice. The verification of the assumptions of univariate normality has been conducted using the procedure for the standardization of the variables, by inspection of a boxplot, and using Shapiro–Wilk’s normality test. Homogeneity of variances was assessed by Levene’s test. A three-way repeated measures ANOVA was run with three independent variables (Gender x Agonistic Experience x Time) and one dependent variable (pain perception at CPT). As the number of participants in the group was balanced, in order to determine the interaction between the variables, Pillai’s criterion was used instead of Wilks’ Lambda, as it is more robust to unequal covariance matrices [54]. Following Cohen [55], partial Eta squared was the measure used to assess effect size (0.01 = small, 0.06 = medium, 0.13 = large). The level of significance was set at *p* < 0.05, while for the testing of multiple univariate interaction effects a Bonferroni adjustment was introduced by dividing the declared level of statistical significance by the number of dependent variables: *p* < 0.025 (i.e., *p* < 0.05 ÷ 2).

## 3. Results

A total of 243 student athletes indicated their willingness to participate in the study. Among these, following a classification conducted through cluster analysis to distinguish a group with more competitive experience from a second group consisting of athletes with less competitive experience, and wanting to balance the number of males and females in the two groups, in the end, 43 student athletes booked on the list did not participate. Therefore, 200 was the final number of participants, 100 male and 100 female, M_age_ = 22.31 SD = 3.33. The distance to the end centres of the cluster = 6.672, Higher Experience group: max distance = 5.045 min = 0.591, Lower Experience Group: max distance = 3.93 min = 0.459. All participants who started the test completed it regularly, therefore, no drop-outs were recorded.

Table 1 below shows the comparison between the group with the lowest competitive experience and the group with the highest competitive experience, considering gender, years of competitive practice, number of training sessions per week, average duration of training sessions and sports practised.

A three-way repeated measures ANOVA (2 X 2 X 2) was conducted to examine both the effects of the membership in the Group of Lower Agonistic Experience vs. Higher Agonistic Experience and Gender of the athletes on the intensity of perceived pain at the beginning of the CPT (L_0_) and after a 90-s interval (L_1_). A residual analysis was performed to test for the assumptions of the three-way repeated measures ANOVA. Outliers were assessed by inspection of a boxplot, normality was assessed using Shapiro–Wilk’s normality test for each cell of the design and homogeneity of variances was assessed by Levene’s test. There were no outliers, residuals were normally distributed (*p* > 0.05) and there was a homogeneity of variances (*p* = 0.697). A statistically significant three-way interaction between Group, Gender and Time resulted, F(1,49) = 9.771, *p* < 0.001. Statistical significance was accepted at the *p* < 0.025 level for simple two-way interactions and simple main effects. There were statistically significant simple two-way interactions between Gender and Time, F(1,49) = 5.423, *p* < 0.025; Group and Time, F(1,49) = 21.488, *p* < 0.025; but none between Gender and Group, F(1,49) = 2.588, *p* = 0.114. With regard to the first interaction, there was a statistically significant simple main effect on pain perception at L_0_ for females, F(1,99) = 93.567, *p* < 0.025. A Bonferroni adjustment was applied. For males, the mean pain perception at L_0_ was 3.95 (SD = 2.49), and for females, it was 4.58 (SD = 1.72). There was a statistically significant mean difference in pain perception between males and females of −0.62795% CI [−0.754, −0.499], *p* < 0.025, η_p_^2^ = 0.302. At L_1_, the simple main effect between the genders was non-significant: *p* = 0.028 i.e. > 0.025.

**Figure 1:**
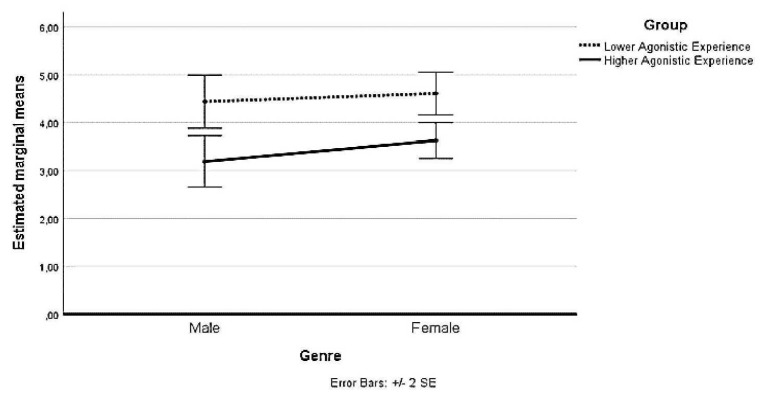
Estimated marginal means of pain perception at Time L_0_.

**Figure 2:**
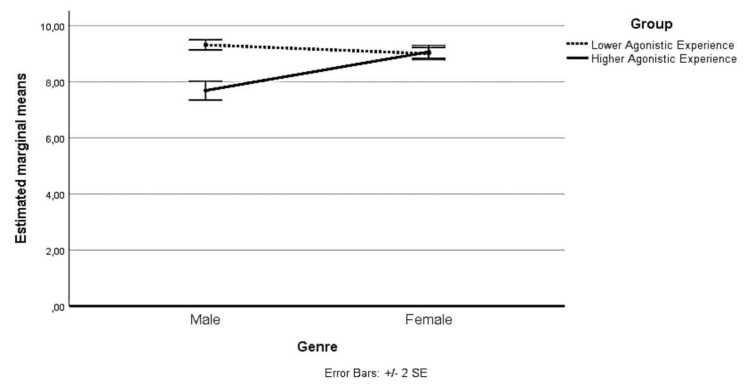
Estimated marginal means of pain perception at Time L_1_.

With regard to the second interaction, at L_0_, no statistically significant simple main effect of Group was reported: F(1,99) = 0.079, *p* > 0.025. At L_1_, a simple significant main effect on pain perception was reported for the Group of Lower Agonistic Experience, F(1,99) = 173.420, *p* < 0.025. For the Group of Lower Agonistic Experience Mean pain perception at L_1_ was 9.33 (SD = 0.47); for the Group of Higher Agonistic Experience, it was 7.79 (SD = 1.43). There was a statistically significant mean difference in pain perception between the groups of 1.534 95% CI [1.303, 1.766], *p* < 0.025, η_p_^2^ = 0.666. The following Table 2 and Figure 1 and Figure 2 show the perceived intensity levels at L_0_ and L_0_ considering the gender and agonistic experience of the participants.

## 4. Discussion

The analysis revealed a significant interaction effect on the ratings of perceived pain at the beginning and end of CPT, attributable in the former case to the gender of participants and in the latter to their agonistic experience. At L_0_, the initial moment of the CPT, females showed a significantly higher perceived pain rating compared to male athletes, regardless of the group they belonged to. This result is consistent with findings in the literature, where the lower threshold of pain in females is supported by ample evidence [56,57,58]. The expansive body of literature in this area suggests that females have lower pain thresholds to a range of pain stimuli when compared to males. Thus, at the moment of first impact with the perception of pain, the assessment of its intensity does not seem to benefit from the support of experience in the modulation of perceived pain. Therefore, only gender-related differences emerge. However, the most interesting aspect of the present study results is what emerged at the end of the CPT, after a 90-s immersion interval of the non-dominant hand in the cold box. It was found that an effect of significantly lower pain perception was associated with the increased competitive experience of male athletes only. Female athletes did not appear to benefit from the experience effect on their pain tolerance. One possible explanation suggests that males are more motivated to tolerate and suppress expressions of pain because of the masculine gender role, whereas the feminine gender role encourages pain expression and produces lower motivation to tolerate pain among females [59]. Other mechanisms have been proposed to explain the differing response to experimental pain between the genders, including hormonal factors, differences in pain modulatory systems and genetic factors. From a more psychosocial perspective, another potential explanation for the gender difference in pain responses involves social role expectancies [60]. Sex hormones also have effects throughout the nervous system, and their plasma concentrations change on a regular basis among both females and males. In addition, hormone levels change in females throughout the menstrual cycle, during pregnancy and after menopause. These differences may have major consequences for pain perception [7]. In females, the pain modulatory system shows menstrual variation with more effect in the ovulatory phase of the cycle compared to the menstrual and luteal phase [61]. Thus, it would seem that after 90 s of CPT, the female athletes felt the physiological effect consisting of a lower inhibition and modulation of pain, resulting in an open admission of a strong increase in pain perception.

A further interpretation could be that the female response of a higher perceived pain, even in the presence of a significant competitive experience, is due to a greater fear of physical damage, otherwise defined as a catastrophic disposition, which, as already reported by several studies, is also relevant in the sporting context [19,21,62,63].

In addition, the influences of different gender-specific coping styles should be considered [9]. These mechanisms also impact pain, and it is suggested that women manage their pain by using more emotionally based and adaptive coping strategies and have a greater propensity to seek emotional support [64]. Conversely, males engage in behavioural distraction, passive coping and avoidance behaviours to cope with pain [65,66]. Therefore, if we consider that the CPT test takes place in a condition of relative and aseptic isolation in the laboratory, this artificial condition may have limited the possibility of using active coping strategies, emotional support seeking and cognitive restructuring that are better adapted to naturalistic and non-laboratory contexts. However, in males, who prefer passive and avoidance coping, CPT delivery may not have interfered with the activation of habitual coping modes and contributed to the control of perceived pain intensity.

## 5. Limitations

The present study should also be considered in light of some additional limitations. The cross-sectional nature of the design and voluntary recruitment could be corrected by a future replication considering a randomized extraction from a larger pool of participating athletes. Given the wide range of sports disciplines included in the study, a replication could instead focus also on the comparison of the perception of pain among athletes of specific sports. In this case, this criterion should be considered when randomising, thus resulting in a homogeneous presence of the disciplines considered in the groups. Considering the peculiarity of the stimulus used in the test (cold), the lack of athletes from winter disciplines (skiing, ice skating, bobsleigh) among the participants could have somehow deprived the study of information on the adaptation response to the CPT test in athletes who are used to managing the cold variable among the environmental conditions in which they usually compete.

A further feature of this work is that the CPT did not include, unlike other studies, a preliminary physical activation phase of the athlete (depending on the discipline practiced), instead the assessment of the algic component took place, so to speak, “coldly”. A further limitation is not having considered the menstrual cycle in the females participating in the study and thus pain modulation under endocrine influence. Indeed, some results have shown that pain sensation is increased during the luteal phase and that high serum progesterone and oestradiol concentrations correspond to lower values for pain intensity [67]. The results should also be considered in the light of the short interval of the testing carried out. The 1.5 min limit was dictated by a precautionary decision, with the intention of guaranteeing extreme safety to the participants in the study and at the same time having a time interval that is considered useful to activate, as indicated by other scholars who suggested that 90 to 120 s of stimulation should be sufficient to achieve a true peak response [68]. In any case, the extension of the CPT to 3 min, carried out in other previous studies [69,70,71], could possibly have affected the process of inhibitory control and modulation of perceived pain in a different way within male and female athletes.

## 6. Study Strengths

In contrast with other studies carried out previously, the detection of the perceived intensity of pain was performed at the initial moment of immersion of the non-dominant hand in the box and after an interval of 90 s. This method provided a way to record the progressive and incremental change in perceived pain intensity in both groups. In previous studies using the CPT method with athletes, it is not specified, whether the assessment of perceived pain intensity was performed at the beginning of the test, after an interval or whether an average measure of perceived intensity between two or more specific intervals was considered. In our opinion, this lack of specification constitutes a limitation of previous works that may have prevented the clarification of aspects of significant differentiation in the processes of coping and modulation of the painful experience.

## 7. Conclusions

In the literature the question of identifying the number and the influence of factors involved in the inhibitory control of pain in male and female athletes remains open. This study was able to demonstrate with a sufficiently large number of participants that the increase in the intensity of pain perceived during cold stimulation by means of the CPT presents in male athletes a significant inhibitory modulation ascribable to factors related to competitive experience accumulated over time. Although there were no significant differences in terms of the total years of practice and the number of weekly training sessions and their duration in the female subsample with more competitive experience, there was no equal advantage for them in the control of pain perception.

## Figures and Tables

**Table 1:** Comparison between the group with lower agonistic experience and the group with higher experience considering gender and sports practised.

						Lower Agonistic Experience						Higher Agonistic Experience						
		N	(%)	ICP	(SD)	WTS	(SD)	ADT	(SD)	ICP	(SD)	WTS	(SD)	ADT	(SD)	P (Experience)		
Gender	Male	100	50.0	5.09	1.83	2.41	1.21	1.51	0.44	9.67	2.43	2.98	1.12	1.71	0.53	<0.001	<0.05	<0.01
	Female	100	50.0	4.50	1.94	2.43	1.20	1.57	0.49	10.34	2.21	3.43	1.16	1.78	0.62	<0.001	<0.001	ns
	Total	200	100%	4.82	1.89	2.42	1.20	1.54	0.46	9.96	2.25	3.18	1.16	1.74	0.57	<0.001	<0.01	ns
	P (gender)			>0.05		>0.05		>0.05		>0.05		>0.05		>0.05				
Sport	Martial arts	11	5.5	5.72	1.91	2.63	1.25	1.27	0.45	9.10	2.30	4.27	1.30	1.45	0.40	<0.001	<0.001	<0.01
	Soccer referee	11	5.5	4.82	1.84	1.63	1.20	1.18	0.48	6.45	2.50	1.90	1.35	1.27	0.55	<0.001	ns	ns
	Athletics (100 m)	4	2.0	3.25	1.92	3.23	1.15	2.00	0.51	10.25	2.35	3.25	1.20	2.00	0.65	<0.001	ns	ns
	Shot put	4	2.0	3.00	1.41	2.00	1.21	1.50	0.42	8.00	2.12	3.00	0.71	2.00	0.71	<0.001	<0.001	<0.001
	Swimming	25	12.5	7.40	2.07	3.40	1.15	1.20	0.40	10.28	2.25	3.60	1.41	1.48	0.55	<0.001	ns	<0.001
	Soccer	34	17.0	6.20	2.26	3.41	1.16	1.35	0.48	11.20	2.40	3.85	1.40	1.50	0.55	<0.001	<0.05	<0.05
	Dance	22	11.0	3.40	1.80	1.95	1.25	1.36	0.52	12.40	2.50	2.77	0.95	1.50	0.65	<0.001	<0.001	ns
	Gymnastics	11	5.5	4.80	2.13	3.27	1.22	1.27	0.44	13.44	2.40	3.63	1.30	1.36	0.45	<0.001	<0.05	ns
	Tennis	10	5.0	5.93	1.90	2.40	1.22	2.10	0.49	14.10	2.15	4.30	1.30	2.50	0.60	<0.001	<0.001	<0.001
	Volleyball	8	4.0	6.23	1.94	2.62	1.18	1.37	0.40	10.25	2.55	2.87	1.30	1.50	0.50	<0.001	ns	<0.05
	Rugby	6	3.0	2.30	1.88	1.82	1.16	1.82	0.46	8.65	2.30	3.15	1.40	2.00	0.50	<0.001	<0.001	<0.01
	Archery	4	2.0	7.00	2.12	1.00	1.23	2.00	0.43	14.00	2.12	2.00	0.71	2.00	0.71	<0.001	<0.001	ns
	Horseback riding	4	2.0	6.00	1.41	2.00	1.22	2.00	0.47	8.00	1.41	4.00	0.71	2.00	0.71	<0.001	<0.001	ns
	Cycling	5	2.5	5.60	1.85	3.00	1.18	1.20	0.50	9.60	2.40	3.20	1.30	2.00	0.55	<0.001	ns	<0.01
	Body Building	4	2.0	4.00	1.41	3.00	1.24	2.00	0.43	9.00	2.12	4.00	0.71	2.00	0.71	<0.001	<0.001	ns
	Power lifting	16	8.0	3.50	2.38	1.50	1.18	1.50	0.45	8.25	2.20	1.50	1.25	1.79	0.50	<0.001	ns	<0.001
	Crossfit	21	10.5	2.82	1.87	2.38	1.25	1.19	0.48	6.38	2.15	2.90	1.35	1.38	0.45	<0.001	<0.01	<0.01
	Total	200	100%	4.82	1.89	2.42	1.20	1.54	0.46	9.96	2.25	3.18	1.16	1.74	0.57	<0.001	<0.01	>0.05

ICP = Years of competitive practice; WTS = Number weekly training sessions; ADT = Average duration of training session; SD = Standard Deviation.

**Table 2:** Pain perception considering Gender, Group and Time.

					Confidence Interval 95 %	
Genre	Group	Time	Mean	Stand. Err.	Lower Bound	Higher Bound
Male	Lower Agonistic Experience	L_0_	4.438	0.278	3.882	4.993
		L_1_	9.313	0.089	9.135	9.490
	Higher Agonistic Experience	L_0_	3.188	0.268	2.652	3.723
		L_1_	7.688	0.169	7.349	8.026
Female	Lower Agonistic Experience	L_0_	4.609	0.223	4.163	5.056
		L_1_	9.000	0.111	8.777	9.223
	Higher Agonistic Experience	L_0_	3.625	0.186	3.354	3.996
		L_1_	9.063	0.115	8.832	9.293

## Data Availability

The datasets during and/or analysed during the current study are available from the corresponding author on reasonable request.
